# Urinary Tryptophan–Kynurenine Pathway Profiling in Bulgarian Children with Autism Spectrum Disorder (ASD): Neopterin Co-Varies with Kynurenine and Quinolinic Acid

**DOI:** 10.3390/metabo16030169

**Published:** 2026-03-04

**Authors:** Victor Slavov, Lubomir Traikov, Stanislava Ciurinskiene, Radka Tafradjiiska-Hadjiolova, Tanya Kadiyska

**Affiliations:** 1 Department of Medical Physics and Biophysics, Medical University of Sofia, 1431 Sofia, Bulgaria; lltraikov@medfac.mu-sofia.bg; 2 Vsiaka Duma Society, 1000 Sofia, Bulgaria; stanislava.ciurinskiene@abv.bg; 3 Department of Physiology and Pathophysiology, Medical University of Sofia, 1431 Sofia, Bulgaria; rhadjiolova@medfac.mu-sofia.bg (R.T.-H.); tkadiyska@medfac.mu-sofia.bg (T.K.); 4 Genetic Medico-Diagnostic Laboratory Genica and Genome Center Bulgaria, 1612 Sofia, Bulgaria

**Keywords:** autism spectrum disorder, urine biomarkers, tryptophan, kynurenine pathway, quinolinic acid, kynurenic acid, neopterin, indoleamine 2,3-dioxygenase (IDO), pediatric metabolomics

## Abstract

**Background/Objectives**: Autism spectrum disorder (ASD) is biologically heterogeneous, and immune-linked variation may be associated with differences in tryptophan–kynurenine pathway (KP) metabolism. Here, we report a targeted urinary profile of KP metabolites, NAD (nicotinamide adenine dinucleotide), and neopterin in a Bulgarian pediatric ASD cohort to describe within-cohort patterns and associations. **Methods**: Second-morning, acid-stabilized spot urine was collected from 73 children with ASD in Bulgaria (3–13 years; 57 males; 16 females). No contemporaneous neurotypical control group was enrolled; therefore, laboratory-provided reference limits are reported only as contextual benchmarks and are not interpreted as ASD-specific abnormalities. Tryptophan (TRP), kynurenine (KYN), kynurenic acid (KYNA), 3-hydroxykynurenine (3-HK), quinolinic acid (QUIN), NAD, and neopterin were quantified and derived indices were computed (KYN/TRP × 1000; QUIN/KYNA). Non-parametric statistics, Benjamini–Hochberg false discovery rate (FDR) correction, and Spearman correlation analyses were applied. **Results**: Neopterin was strongly associated with QUIN and KYN in creatinine-normalized data (QUIN: ρ = 0.59, q36 = 2.64 × 10^−7^; KYN: ρ = 0.54, q36 = 3.69 × 10^−6^); these associations persisted when reconstructed as absolute concentrations (e.g., QUIN_abs: ρ = 0.68, q36 = 2.69 × 10^−10^) and after partial Spearman correlation controlling for spot creatinine (partial ρ = 0.46, q = 2.52 × 10^−4^). One NAD value was <LOQ and was imputed as ½LOQ; sensitivity analyses did not materially change inference. **Conclusions**: In this ASD-only cross-sectional dataset, urinary neopterin levels co-varied with urinary KYN and QUIN and with KP indices. Clinical interpretation and causal inference require controlled and longitudinal studies with richer covariate capture.

## 1. Introduction

Autism spectrum disorder (ASD) shows substantial biological heterogeneity, and evidence from metabolomics and immunology suggests that a subset of individuals exhibits coordinated immune–metabolic alterations, often alongside gastrointestinal comorbidities [1,2]. Accessible biofluid readouts may therefore help to stratify ASD subgroups and to connect immune signaling to specific metabolic pathways.

Tryptophan (TRP) metabolism is of particular interest because immune-regulated diversion of TRP into the kynurenine pathway (KP) can alter the balance between neuroactive metabolites [3]. Indoleamine 2,3-dioxygenase (IDO1/IDO2) catalyzes the first and rate-limiting step of TRP degradation toward kynurenines and is inducible by cytokines, especially interferon-γ (IFN-γ) [3]. Downstream, kynurenic acid (KYNA) modulates excitatory neurotransmission and is often discussed as relatively protective, whereas quinolinic acid (QUIN) is an N-methyl-D-aspartate receptor (NMDAR) agonist with excitotoxic and pro-oxidant mechanisms [4,5]. Accordingly, higher QUIN relative to KYNA has been linked to neuroinflammatory and neuropsychiatric phenotypes [4,6].

Several metabolomics and targeted studies have reported altered TRP–KP metabolism in ASD, including changes in KYN-related metabolites and ratios reflecting IDO index, although results across cohorts differ [2,7,8,9]. Neopterin, a pteridine produced by activated monocytes/macrophages in response to IFN-γ, is a well-established marker of cellular immune activation and oxidative stress [10,11]. Because IFN-γ can concurrently promote neopterin production and induce IDO-mediated TRP catabolism, joint assessment of urinary neopterin and KP metabolites provides an opportunity to examine whether neopterin co-varies with KP markers within the cohort [3,10].

Evidence on urinary pterins (neopterin/biopterin) in ASD is limited and heterogeneous, with early small studies reporting conflicting directions of change across cohorts [12]. For this reason, we emphasize within-cohort association patterns and robustness checks across data representations, rather than case-control inference. This variability underscores biological heterogeneity and highlights the need for age-matched controls and careful contextualization when reference limits are used.

In this study, we report quantitative urinary data for nine markers (KP metabolites, NAD (nicotinamide adenine dinucleotide), and neopterin, together with derived indices reflecting IDO index (KYN/TRP × 1000) and QUIN/KYNA balance) in 73 Bulgarian children with ASD. Our primary objective is to describe within-cohort distributions and co-variation patterns and to test whether neopterin co-varies with KP markers across multiple data representations. Because no contemporaneous neurotypical control group was enrolled, laboratory-provided reference limits are presented strictly as contextual benchmarks and are not used for case-control inference.

## 2. Materials and Methods

### 2.1. Study Design and Participants

This cross-sectional study included 73 children with ASD from Bulgaria (age range 3–13 years; median age and IQR: 5 (4–7); 57 males and 16 females). ASD diagnoses were ascertained from specialist clinical records (epicrises) available to the research team. The source documentation includes DSM-IV-TR terminology for a substantial proportion of participants (reflecting the timing of the original diagnoses and local documentation practices) and also includes DSM-5 terminology where present [13,14]. No contemporaneous neurotypical control group was enrolled; analyses therefore focus on within-cohort patterns and associations.

### 2.2. Ethics

The study was approved by the Research Ethics Committee of University Hospital “St. Ivan Rilski”, Sofia, Bulgaria (protocol number 3/26.04.2023). Written informed consent was obtained from the parents/guardians of all participants after full explanation of the study purposes and procedures.

### 2.3. Urine Collection and Pre-Analytical Standardization

Urine specimens were collected at home using an acid-stabilized urine collection kit (T928; biovis Diagnostik MVZ GmbH, Limburg, Germany) used within the provider’s Item 928 second-morning urine workflow and intended for second-morning, midstream sampling. This second-morning, acid-stabilized approach (collection 2–4 h after the first morning void) aligns with protocols previously described in peer-reviewed studies using the same provider-based urine metabolite profiling workflow [15].

Caregivers were instructed to collect the second-morning void approximately 2–4 h after the first-morning urine (minimum 2 h), to avoid excessive fluid intake before sampling, and to refrain from strenuous physical activity on the morning of collection. They were also instructed to avoid caffeine-containing beverages and energy drinks for at least 16 h prior to collection and to report any acute febrile illness at the time of sampling. To minimize acute confounding when feasible, caregivers were asked to avoid non-essential dietary supplements; ongoing clinically indicated prescription treatments and medically required diets were not discontinued for study purposes. According to the caregiver report, no participant had an acute infection or febrile illness and no NSAIDs/anti-inflammatory medications or tryptophan-/NAD-precursor supplements were used during the two weeks preceding urine collection (0/73 for each). Compliance with pre-analytical instructions was based on caregiver report and was not independently verified.

Immediately after voiding into a clean collection container, a midstream aliquot was transferred into the kit tube containing a pre-inserted stabilizer plate to achieve acid stabilization. Acidification was verified using the provided pH strip per kit instructions; samples not meeting the required acidification criterion were recollected on a different day. Stabilized samples were stored at ambient temperature per kit instructions (10–30 °C) and returned to the analytical laboratory in the provided transport container. Shipments were made on working days only (avoiding Friday dispatch, weekends, and public holidays), and samples were not deposited in mailboxes to minimize transit delays and uncontrolled storage conditions.

### 2.4. Laboratory Analyses

Outsourced laboratory analyses. Targeted urinary analytes were measured as a fee-for-service by an external clinical laboratory, biovis Diagnostik MVZ GmbH (65552 Limburg a. d. Lahn-Eschhofen, Germany), accredited by the DAkkS (German Accreditation Body) in accordance with DIN EN ISO 15189 (Accreditation Certificate D-ML-13188-01-00) [16]. Participant recruitment and pre-analytical procedures (second-morning midstream urine collection, acid stabilization using the provider’s collection kit, storage, and shipment logistics) were coordinated via the laboratory Genome Center Bulgaria (1612 Sofia, Bulgaria); samples were shipped to the German provider laboratory for analytical measurement and report release. Quantitative determination of TRP, KYN, KYNA, 3-HK, QUIN, NAD, and neopterin was performed by targeted LC–MS/MS under the provider’s validated procedures within its ISO 15189 quality management system.

For methodological transparency, the service laboratory provided the following LC–MS/MS method details for the targeted panel. LC–MS grade water (Sigma-Aldrich, St. Louis, MO, USA) and methanol (Honeywell, Charlotte, NC, USA) were used, with formic acid (>99.9%, Sigma-Aldrich) and trifluoroacetic acid (>99.5%, Sigma-Aldrich) as mobile phase modifiers. Chromatographic separation was performed on an Agilent Infinity 1290 LC system with autosampler (Agilent Technologies, Santa Clara, CA, USA) using a Restek Raptor ARC-18 column (2.7 μm, 100 mm × 2.1 mm; Restek, Bellefonte, PA, USA). Injection volume was 8 μL. Mobile phase A was water with 0.15% formic acid and 0.01% TFA; mobile phase B was methanol with 0.15% formic acid and 0.01% TFA. Flow rate was 0.4 mL/min. The gradient was: 97% A (0–0.65 min), 85% A (0.65–1.3 min), 35% A (1.3–3.0 min), 20% A (3.01–5.0 min), and re-equilibration to 97% A (5.0–7.5 min). Calibration was matrix-matched and performed using a four-point scheme with linear regression within the reported analyte-specific ranges.

MS detection used a SCIEX TripleQuad 5500+ triple-quadrupole (SCIEX, Framingham, MA, USA) in scheduled multiple reaction monitoring (MRM) mode. Positive ESI was used for all analytes except quinolinic acid, which was measured in negative ESI. Source conditions were: ESI+ (CUR 35; CAD 8; IS 5500; TEM 500; GS1 62; GS2 60) and ESI− (CUR 35; CAD 8; IS −4500; TEM 500; GS1 62; GS2 60). Quantification used analyte-specific isotopically labeled internal standards: tryptophan-d5, kynurenine-d3, 3-hydroxykynurenine-d4, kynurenic acid-d5, quinolinic acid-d3, and (NAD+)-13C5. Isotopically labelled internal standards were sourced from Sigma-Aldrich (tryptophan-d5, kynurenine-d3, kynurenic acid-d5, quinolinic acid-d3), Buchem B.V., Apeldoorn, The Netherlands (3-hydroxykynurenine-d4), and Cambridge Isotope Laboratories, Inc., Tewksbury, MA, USA ((NAD+)-13C5). The MRM transitions, declustering potentials, collision energies, and retention times for TRP, KYN, 3-HK, KYNA, QUIN, and NAD are provided in Table S1.

Spot urinary creatinine was measured by a routine enzymatic method (UV/VIS photometric assay) under the same quality system. Samples were processed in standardized assay batches; the laboratory reported no analytical drift across batches. Testing was ordered under the provider’s Neurotransmitter/NT-Tryptophan service (Item no. 928). As part of ISO 15189-compliant routine operations, the provider performs method validation, calibration, and internal quality control according to its laboratory procedures. Quality controls included ClinCheck^®^ urine controls (biogenic amines, L1 and L2;RECIPE Chemicals + Instruments GmbH, Munich, Germany); for analytes without certified QC material, target ranges were established via controlled spiking and dilution experiments. The laboratory provided a validation summary including analyte-specific LOD and LOQ (defined as 3× and 6× S/N), linear ranges, and intra-assay precision (CV%) for the analytes reported here (Table S2), and stated that the assay was internally validated in accordance with CLSI guidance (including linearity, precision, recovery, and matrix-effect evaluation). Results were returned as creatinine-normalized concentrations (μmol/g creatinine for TRP, KYN, KYNA, 3-HK, QUIN, and neopterin; nmol/g creatinine for NAD) and as derived ratio indices defined below (computed variables rather than direct measurements of enzyme activity). In particular, the IDO index (KYN/TRP × 1000) is a ratio-based estimate and not a direct measure of indoleamine 2,3-dioxygenase enzyme activity.

For additional context, the provider’s targeted LC–MS/MS workflow for the same service has been described in a published report [15]. That report documents the UHPLC platform and the general use of isotopically labeled internal standards. In the present study, the method-specific parameters used for our samples (LC conditions, source settings, and analyte-specific MRM transitions) are reported above and in Table S1, based on the service laboratory’s method summary.

### 2.5. Reference Values and Data Handling

Reference limits (lower/upper) were taken from the commercial documentation accompanying the analytical service and are defined for the same second-morning, acid-stabilized urine collection and reporting units used here (creatinine-normalized μmol/g creatinine for TRP, KYN, KYNA, 3-HK, QUIN, and neopterin; nmol/g creatinine for NAD). The provider applies these limits across the pediatric age range served by the test. In a published description of the provider’s workflow, reference ranges are derived from percentile distributions in self-reported healthy individuals and are continuously checked/optimized using an expanded internal sample database (>10,000 samples) [15]. These limits are reported here only as contextual benchmarks. They may not be tightly age- or geography-matched to Bulgarian children. Therefore, they are not used for case-control inference in this study.

Spot urine samples with creatinine below the laboratory-provided lower reference limit (400 mg/L) were treated as potentially dilute; primary analyses were performed on the full dataset, and sensitivity analyses excluded these samples to assess the robustness of creatinine normalization [17,18]. This dilution-screening approach is consistent with published use of the same provider workflow, where creatinine is used to contextualize urine concentration and samples outside the creatinine reference range are excluded from statistical analyses [15].

For NAD, one sample was reported as below the limit of quantification (“<25 nmol/g creatinine”); this left-censored value was imputed as 12.5 nmol/g creatinine (half the limit of quantification), a standard approach when the proportion of censored observations is small.

### 2.6. Statistical Analysis

Analyses were performed in Python (v3.11) using SciPy and statsmodels. Continuous variables are summarized as median (interquartile range, IQR) due to non-normal distributions. Between-group comparisons (e.g., by sex or by neopterin status) used the Mann–Whitney U test with effect sizes reported as Cliff’s delta (δ). Associations among markers were assessed using Spearman’s rank correlation (ρ). Multiple testing was controlled using the Benjamini–Hochberg false discovery rate (FDR) procedure [19], applied separately within each pre-specified analysis family. For correlation analyses (Figure S1; Table S5), q36 denotes BH-FDR-adjusted *p*-values across the 36 unique pairwise correlations of the full 9-variable panel. The 36 correlations arise from the 9-variable panel as 9 choose 2 (9 × 8/2) unique pairwise comparisons. The q36 family comprised TRP, KYN, KYNA, 3-HK, QUIN, NAD, neopterin, IDO index, and QUIN/KYNA. For neopterin-group comparisons, FDR correction was applied across 8 tests, and for sex-group comparisons across 10 tests. These family sizes correspond to the number of outcome-level tests performed within each pre-specified group-comparison block. No additional adjustment across families was applied because families reflect distinct pre-specified analytic questions; inference is therefore made within-family. Specifically, the q8 family comprised TRP, KYN, 3-HK, QUIN, KYNA, NAD, IDO index, and QUIN/KYNA, whereas the q10 family comprised TRP, KYN, KYNA, 3-HK, QUIN, NAD, IDO index, QUIN/KYNA, neopterin, and creatinine. Statistical significance was set at q < 0.05 (two-sided tests), where q denotes the FDR-adjusted *p*-value. To probe potential shared-denominator effects from creatinine normalization, absolute concentrations were reconstructed as C_abs = C_norm × creatinine (g/L). Creatinine was converted from mg/L to g/L prior to reconstruction. Key neopterin–KP associations were repeated in reconstructed absolute units and as a rank-based partial correlation controlling for spot creatinine. For partial correlations, variables were rank-transformed, residualized on ranked creatinine using simple linear regression, and Pearson correlation was computed on residuals (df = *n* − 3). The same rank-based partial correlation approach was used for partial correlations controlling for age (Table S7). As an additional covariate-adjusted check, rank-based linear regression models (OLS on z-scored ranks) were fit in reconstructed absolute units to evaluate neopterin_abs associations with QUIN_abs and KYN_abs while adjusting for age and sex; 95% confidence intervals were obtained by bootstrap resampling (1000 iterations). Analyses explicitly labeled as exploratory (e.g., age-related analyses in Table S7) report nominal *p*-values and are interpreted cautiously. For correlation heatmaps, stars denote FDR-corrected significance (* q36 < 0.05; ** q36 < 0.01; *** q36 < 0.001).

## 3. Results

### 3.1. Cohort Characteristics and Sample Integrity

The cohort comprised 73 children with ASD (57 males and 16 females; age range 3–13 years; median age and IQR: 5 (4–7)). Urinary creatinine concentration was used as a dilution marker for spot samples. Creatinine was below the laboratory-provided lower reference limit (400 mg/L) in 11/73 samples (15.1%). Participant characteristics and analytical notes are summarized in Table 1.

### 3.2. Urinary Marker Distributions with Laboratory Reference Limits

**Figure 1:**
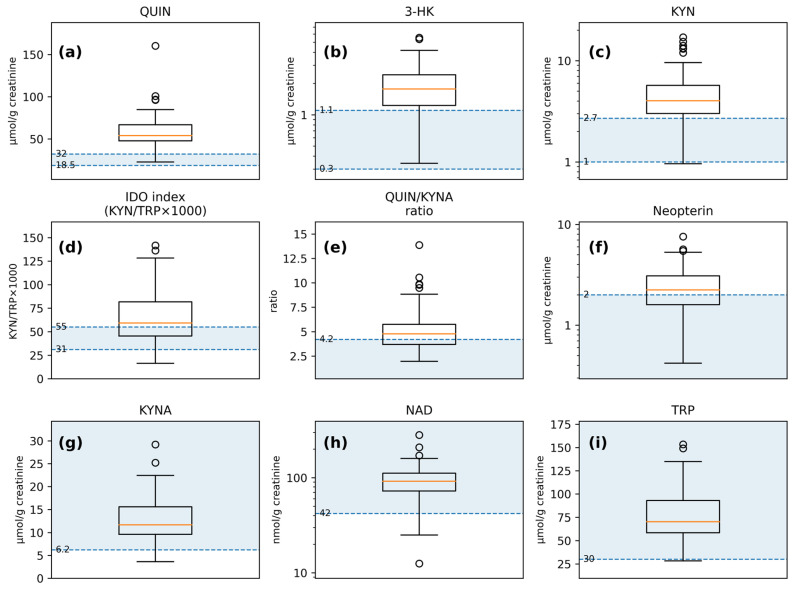
Distributions of urinary markers shown against laboratory-provided reference intervals and decision limits: (**a**) QUIN; (**b**) 3-HK; (**c**) KYN; (**d**) IDO index (KYN/TRP × 1000); (**e**) QUIN/KYNA ratio; (**f**) Neopterin; (**g**) KYNA; (**h**) NAD; (**i**) TRP. Shaded bands/lines denote reference intervals or one-sided thresholds; where reference information is one-sided, only the corresponding bound is shown. Boxplots depict Q1–Q3 (IQR) with the median as the center line; whiskers extend to the most extreme values within 1.5 × IQR; points denote outliers.

Distributions of urinary markers are shown in Figure 1, and summary statistics are provided in Table 2. Because the study lacks matched neurotypical controls, laboratory reference limits are presented for context only and are not interpreted as ASD-specific abnormalities. Subsequent analyses therefore focus on within-cohort associations.

### 3.3. Correlation Structure of KP Markers and Neopterin

**Figure 2:**
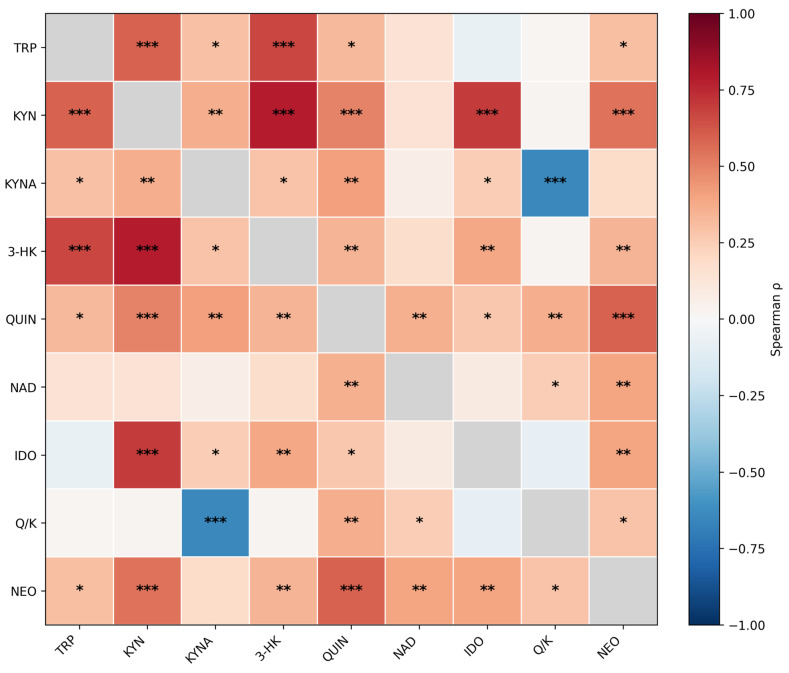
Spearman correlation heatmap of the 9-variable urinary marker panel (TRP, KYN, KYNA, 3-HK, QUIN, NAD, neopterin, IDO index, and QUIN/KYNA; *n* = 73) based on creatinine-normalized concentrations (μmol/g or nmol/g creatinine) and derived indices. Cell color represents Spearman’s ρ (full matrix shown). False discovery rate (FDR) significance is indicated by stars (* q36 < 0.05; ** q36 < 0.01; *** q36 < 0.001), where q36 denotes Benjamini–Hochberg–adjusted *p*-values across the 36 unique pairwise correlations of the 9-variable panel. Cells are labeled with significance stars only (no numeric ρ values); numeric Spearman ρ values are provided in Figure S1. Robustness checks addressing potential shared-denominator effects from creatinine normalization (reconstructed absolute concentrations and partial correlations adjusting for spot creatinine) are reported in Table S5.

Spearman correlation analysis revealed a consistent positive correlation block among TRP, KYN, 3-HK, QUIN and neopterin, alongside expected structure across KP indices (Figure 2). Neopterin showed strong positive associations with QUIN (ρ = 0.59, q36 = 2.64 × 10^−7^) and KYN (ρ = 0.54, q36 = 3.69 × 10^−6^), and moderate associations with IDO index (ρ = 0.39, q36 = 0.002) and the QUIN/KYNA ratio (ρ = 0.28, q36 = 0.026).

These relationships were preserved in a sensitivity analysis excluding dilute spot samples (creatinine <400 mg/L), where neopterin remained strongly associated with QUIN (ρ = 0.55, nominal *p* = 4.01 × 10^−6^). Because this exclusion analysis is a robustness check rather than a separate discovery screen, we report the effect size and nominal *p*-value; multiple-testing control (q36) is applied to the primary full-cohort analyses.

To evaluate potential shared-denominator effects from creatinine normalization, we reconstructed absolute concentrations (μmol/L or nmol/L) from creatinine-normalized values and spot creatinine and repeated correlations. Neopterin remained strongly associated with QUIN in absolute units (ρ = 0.68, q36 = 2.69 × 10^−10^) and after partial adjustment for spot creatinine (partial ρ = 0.46, q36 = 2.52 × 10^−4^; Table S5). In an age- and sex-adjusted rank-based linear model using reconstructed absolute units, neopterin_abs remained strongly associated with QUIN_abs (standardized β = 0.68; 95% bootstrap CI 0.51–0.80; *p* = 6.13 × 10^−11^) and with KYN_abs (standardized β = 0.60; 95% bootstrap CI 0.41–0.74; *p* = 1.69 × 10^−8^) (Table S8). A full correlation heatmap with numeric Spearman ρ values in cells is provided in Figure S1. For readability, Figure 2 shows significance stars only (q36), while numeric ρ values are provided in Figure S1.

### 3.4. Exploratory Stratification by Provider Neopterin Threshold (Dilution-Sensitive)

**Figure 3:**
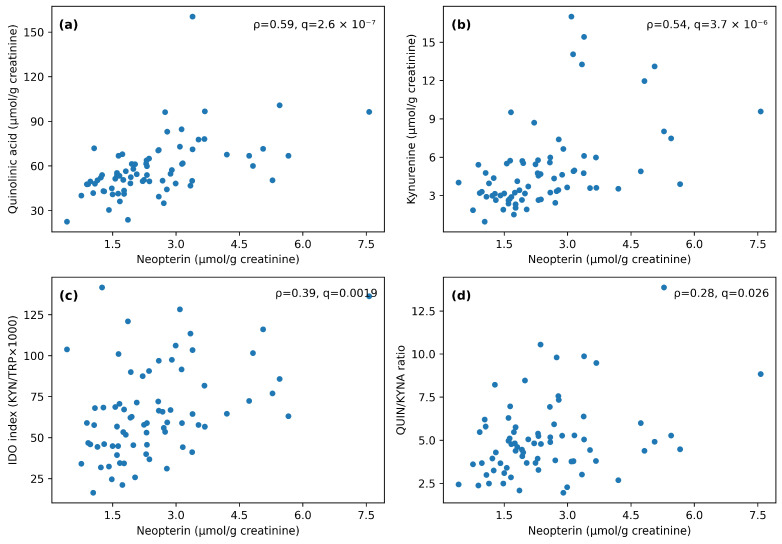
Scatterplots illustrating associations between urinary neopterin and select kynurenine-pathway markers and indices (*n* = 73). (**a**) Neopterin vs. quinolinic acid (QUIN); (**b**) Neopterin vs. kynurenine (KYN); (**c**) Neopterin vs. IDO index (KYN/TRP × 1000); (**d**) Neopterin vs. QUIN/KYNA ratio. Each dot represents one participant; Spearman’s ρ and the corresponding significance metrics displayed in the panels are reported for each association.

To explore whether children above the provider’s neopterin cut-off also show a broader shift in creatinine-normalized KP metabolites, we stratified participants by neopterin status. To visualize dilution-sensitive contrasts, participants were stratified by provider neopterin threshold (>2 μmol/g creatinine, *n* = 40 vs. ≤2 μmol/g creatinine, *n* = 33). This stratification is exploratory; inference relies on continuous associations and on analyses that address urine dilution (reconstructed absolute concentrations and creatinine-adjusted correlations). In creatinine-normalized units, the above-reference neopterin group showed higher TRP, KYN, 3-HK, QUIN, IDO index (KYN/TRP × 1000), and NAD compared with the within-reference group (all q < 0.05 after FDR correction), whereas QUIN/KYNA ratio and KYNA were not significant after FDR correction (q ≥ 0.05; Table 3). However, the above-referenced group also had lower spot creatinine (median 717.00 (502.75–923.25) vs. 925.00 (652.00–1320.00) mg/L; *p* = 0.022, q = 0.154), and reconstructed absolute concentrations (μmol/L or nmol/L) did not differ significantly between groups after FDR correction (Table S6). Accordingly, subgroup contrasts should be interpreted cautiously in the context of urine dilution and shared-denominator effects, whereas continuous neopterin–KP associations remained consistently positive in absolute and creatinine-adjusted analyses (Table S5). Figure 3 highlights representative associations.

### 3.5. Sex-Stratified Exploratory Analyses

Exploratory comparisons between males and females did not reveal robust sex differences after BH-FDR correction across the 10-test family (q10). KYNA showed a nominal sex difference (*p* = 0.012), but this did not survive multiple-testing adjustment (q = 0.115; Table S4).

### 3.6. Age-Related Exploratory Analyses

Age was inversely associated with neopterin (Spearman ρ = −0.37, *p* = 1.14 × 10^−3^) and QUIN (ρ = −0.47, *p* = 3.26 × 10^−5^). Partial Spearman correlations controlling for age indicated that the neopterin–QUIN association remained positive and significant after age adjustment (partial ρ = 0.50, *p* = 5.24 × 10^−6^), as did the neopterin–KYN association (partial ρ = 0.53, *p* = 1.69 × 10^−6^). Full results are provided in Table S7.

## 4. Discussion

In this Bulgarian pediatric ASD cohort, urinary neopterin co-varied consistently with QUIN and KYN across creatinine-normalized values, reconstructed absolute concentrations, and creatinine-adjusted analyses. Because no contemporaneous neurotypical control group was enrolled, laboratory-provided reference limits are reported only as contextual benchmarks and are not interpreted as ASD-specific abnormalities. Accordingly, the primary contribution of this work is the within-cohort correlation structure and neopterin-linked KP patterning, rather than inference about departures from normative pediatric ranges.

Urinary targeted profiling of the tryptophan–kynurenine pathway (KP) in pediatric ASD remains heterogeneous across studies in terms of matrices (urine vs. plasma/serum), normalization strategies, and covariate capture [1,2,7,8,9]. In this ASD-only cross-sectional dataset, the main contribution is not case-control inference but the internally consistent co-variation between neopterin and KP markers (KYN and QUIN), which remained evident across creatinine-normalized reporting, reconstructed absolute units, and creatinine-adjusted analyses. These within-cohort patterns can serve as a starting point for formulating hypotheses to be tested in future controlled, longitudinal studies.

Urinary neopterin was interpreted as a marker of cellular immune activation and oxidative stress [10,11]. Within this ASD cohort, neopterin showed strong positive associations with QUIN and KYN, as well as with KP indices (IDO index and QUIN/KYNA). These associations remained strong when expressed as reconstructed absolute concentrations and after adjustment for spot creatinine (Table S5). This hypothesis-generating pattern is compatible with prior work linking interferon-γ–related pathways to neopterin production and with enhanced tryptophan catabolism via indoleamine 2,3-dioxygenase, although cytokines were not measured in the present study [3,10]. However, in the absence of cytokine measurements, we cannot test whether IFN-γ or other cytokines drive the observed co-variation in this cohort.

Pre-analytical standardization is critical for urinary metabolomics. We used an acid-stabilized second-morning midstream protocol with timing and hydration guidance, and creatinine served as a dilution marker. Nonetheless, 11/73 samples (15.1%) were below the commercial creatinine lower limit, which may inflate creatinine-normalized analyte values [17,18]. Sensitivity analyses excluding these potentially dilute samples retained the key KP–neopterin associations (e.g., neopterin–QUIN ρ = 0.55, nominal *p* = 4.01 × 10^−6^), supporting that the observed relationships are not solely artifacts of urine dilution (Note S1).

We also observed age-related trends, with older children showing lower urinary neopterin and QUIN. This suggests that age-specific pediatric reference standards may be important. Using broad pediatric cut-offs could overestimate the proportion of “above-reference” results in younger participants. Importantly, the neopterin–QUIN association remained significant after age adjustment, supporting that the within-cohort co-variation is not explained by age alone.

To test whether the neopterin–KP co-variation observed here is ASD-specific, future studies should include contemporaneous neurotypical controls recruited from the same catchment area and processed under identical pre-analytics. Controls should be age- and sex-matched, or these variables should be pre-specified covariates. Longitudinal sampling (at least two time-points) would help distinguish stable coupling from transient immune activation. Key covariates should be captured where feasible, including recent infection/fever, medication or supplement exposure, and urine dilution markers (spot creatinine). Detailed medication classes and GI comorbidity data were not captured in a structured form suitable for summary reporting; therefore, residual confounding cannot be fully excluded.

## 5. Conclusions

In a Bulgarian pediatric ASD cohort profiled with a targeted urinary KP panel, neopterin co-varied consistently with KYN and QUIN and remained evident across creatinine-normalized reporting, reconstructed absolute concentrations, creatinine-adjusted analyses, and age/sex-adjusted models. These internally coherent patterns support the use of urinary neopterin as a contextual covariate when describing KP variation within ASD cohorts, while emphasizing that clinical interpretation and causality require matched controls, longitudinal sampling, and richer phenotyping. Future studies integrating urinary KP profiling with inflammatory markers (e.g., IFN-γ) and standardized dilution assessment may clarify the biological and clinical relevance of neopterin-linked KP variation.

## Figures and Tables

**Table 1:** Participant characteristics and analytical notes (age summary statistics: 5 (4–7) years).

Characteristic	Value
Participants, *n*	73
Age range, years	3–13
Age, years, median (IQR)	5 (4–7)
Male, *n* (%)	57 (78.1)
Female, *n* (%)	16 (21.9)
Urinary creatinine (mg/L), median (IQR)	795 (586–976)
Creatinine below 400 mg/L, *n* (%)	11 (15.1)
NAD < 25 nmol/g creatinine (below LOQ), *n* (%)	1 (1.4)

**Table 2:** Urinary kynurenine-pathway markers, indices and neopterin relative to laboratory-provided reference limits (context only) in the autism spectrum disorder (ASD) cohort (*n* = 73).

Analyte	Unit	Reference Limit	Median (IQR)	Below Ref *n* (%)	Above Ref *n* (%)
Quinolinic acid	μmol/g creatinine	18.5–32.0	53.92 (47.78–66.85)	0 (0.0)	70 (95.9)
Kynurenine	μmol/g creatinine	1.0–2.7	4.02 (3.01–5.71)	1 (1.4)	59 (80.8)
3-OH-Kynurenine	μmol/g creatinine	0.3–1.1	1.77 (1.23–2.43)	0 (0.0)	59 (80.8)
IDO index (KYN/TRP × 1000)	–	31–55	59.2 (45.4–81.7)	4 (5.5)	46 (63.0)
QUIN/KYNA ratio	–	<4.2	4.77 (3.69–5.75)	0 (0.0)	45 (61.6)
Neopterin	μmol/g creatinine	<2.0	2.24 (1.60–3.09)	0 (0.0)	40 (54.8)
Kynurenic acid	μmol/g creatinine	>6.2	11.69 (9.61–15.59)	4 (5.5)	0 (0.0)
NAD	nmol/g creatinine	>42	91.7 (72.4–111.6)	4 (5.5)	0 (0.0)
Tryptophan	μmol/g creatinine	>30	70.35 (58.47–92.98)	1 (1.4)	0 (0.0)

Note: Reference limits are laboratory-provided and used as contextual benchmarks (no neurotypical control group). Provider limits may not be age- or geography-matched to Bulgarian children; therefore, ‘above reference’ proportions are descriptive only and do not imply ASD specificity. For one-sided limits (< or >), only the indicated direction is considered outside the reference. Abbreviations: TRP, tryptophan; KYN, kynurenine; KYNA, kynurenic acid; 3-HK, 3-hydroxykynurenine; QUIN, quinolinic acid; NAD, nicotinamide adenine dinucleotide; IDO index = KYN/TRP × 1000. For example, KYNA has a lower threshold (>6.2), so values below 6.2 are counted as below reference; TRP (>30) and NAD (>42) are interpreted analogously.

**Table 3:** Creatinine-normalized group comparisons by neopterin status (Mann–Whitney U; Benjamini–Hochberg false discovery rate (FDR)). q denotes FDR-adjusted *p*-values; Cliff’s δ ranges from −1 to 1, with values farther from 0 indicating greater separation between groups. Units: μmol/g creatinine unless stated; NAD in nmol/g creatinine; ratios are dimensionless. For reconstructed absolute concentrations and spot creatinine comparisons, see Table S6.

Marker	Median (Neopterin High)	Median (Neopterin Normal)	*p*	q (FDR)	Cliff’s δ
Kynurenine	4.91	3.16	2.93 × 10^−5^	1.17 × 10^−4^	0.57
Quinolinic acid	61.65	48.28	1.66 × 10^−5^	1.17 × 10^−4^	0.59
3-OH-Kynurenine	2.1	1.35	0.001	0.003	0.44
Tryptophan	75.88	66.81	0.009	0.013	0.36
IDO index	66.05	51.8	0.01	0.013	0.35
NAD	95.55	74.5	0.008	0.013	0.36
QUIN/KYNA ratio	4.98	4.3	0.056	0.064	0.26
Kynurenic acid	12.75	11.14	0.142	0.142	0.2

## Data Availability

De-identified individual-level data supporting the findings of this study are provided in Table S3. Additional metadata (e.g., exact birthdate, clinical covariates) are not publicly available due to ethical and data-protection constraints. Such data may be made available upon reasonable request to the corresponding author, subject to the required institutional approvals and consistency with participant consent; requests should include a brief research purpose/analysis plan and documentation of the requester’s institutional ethics approval or exemption.

## Supplementary Materials

The following supporting information can be downloaded at: https://www.mdpi.com/article/10.3390/metabo16030169/s1, Table S1: LC–MS/MS MRM transitions and parameters provided by the service laboratory (SCIEX TripleQuad 5500+, scheduled MRM); Table S2. Analytical performance summary provided by the service laboratory (LOD/LOQ, linear range, intra-assay precision); Figure S1: Spearman correlation heatmap (color = ρ; full matrix shown) with numeric Spearman ρ values in cells (*n* = 73). Correlations were computed on creatinine-normalized urinary concentrations (μmol/g creatinine; NAD in nmol/g creatinine) and derived indices (IDO index = KYN/TRP × 1000; QUIN/KYNA); Table S3: De-identified individual urinary marker values with key covariates (age at sampling, sex, spot creatinine); sequential participant IDs; *n* = 73; Table S4: Sex-stratified comparisons (Mann–Whitney U; false discovery rate (FDR)). q denotes BH-FDR-adjusted *p*-values across the variables listed in this table; Table S5: Neopterin–KP associations in creatinine-normalized ratios vs. reconstructed absolute concentrations, including partial correlations adjusting for creatinine; Table S6: Neopterin status subgroup comparisons in reconstructed absolute units and spot creatinine; Table S7: Exploratory age–marker associations (Spearman; partial Spearman controlling age); Table S8: Age- and sex-adjusted rank-based linear models in reconstructed absolute units (OLS on z-scored ranks; bootstrap 95% CI, 1000 resamples); Figure S2: Age associations for urinary neopterin and QUIN (*n* = 73); Note S1: Sensitivity analyses excluding potentially dilute creatinine samples.

## Abbreviations

The following abbreviations are used in this manuscript:

3-HK	3-hydroxykynurenine
ASD	autism spectrum disorder
BH-FDR	Benjamini–Hochberg false discovery rate
CI	confidence interval
DAkkS	German Accreditation Body (Deutsche Akkreditierungsstelle)
DIN	German Institute for Standardization (Deutsches Institut für Normung)
DSM-IV-TR	Diagnostic and Statistical Manual of Mental Disorders, Fourth Edition, Text Revision
EN	European Standard (European Norm)
FDR	false discovery rate
GMDL	Genetic Medico-Diagnostic Laboratory
IDO	indoleamine 2,3-dioxygenase
IDO1	indoleamine 2,3-dioxygenase 1
IDO1/IDO2	indoleamine 2,3-dioxygenase 1 and 2
IDO2	indoleamine 2,3-dioxygenase 2
IFN-γ	interferon-gamma
IQR	interquartile range
ISO 15189	medical laboratory accreditation standard (ISO 15189)
KP	kynurenine pathway
KYN	kynurenine
KYN/TRP	kynurenine-to-tryptophan ratio
KYNA	kynurenic acid
LC–MS/MS	liquid chromatography–tandem mass spectrometry
LOQ	limit of quantification
MRM	multiple reaction monitoring
MVZ	medical care center (German: Medizinisches Versorgungszentrum)
NAD	nicotinamide adenine dinucleotide
NMDAR	N-methyl-D-aspartate receptor
OLS	ordinary least squares
QUIN	quinolinic acid
QUIN/KYNA	quinolinic acid-to-kynurenic acid ratio
SD	standard deviation
TRP	tryptophan
UHPLC	ultra-high-performance liquid chromatography
UV/VIS	ultraviolet/visible
