# Supplementary material for: Urinary Tryptophan–Kynurenine Pathway Profiling in Bulgarian Children with Autism Spectrum Disorder (ASD): Neopterin Co-Varies with Kynurenine and Quinolinic Acid
Source: Metabolites. 2026 Mar 4;16(3):169. doi: 10.3390/metabo16030169 (PMC13027834; doi:10.3390/metabo16030169)
Supplement: Supplementary file 1 [file metabolites-16-00169-s001.zip › metabolites-4171722-supplementary.pdf]

Supplementary Materials: The following supporting information can be downloaded at: https://www.mdpi.com/article/10.3390/metabo16030169/s1, Table S1: LC–MS/MS MRM transitions and parameters provided by the service laboratory (SCIEX TripleQuad 5500+, scheduled MRM); Table S2. Analytical performance summary provided by the service laboratory (LOD/LOQ, linear range, intra-assay precision). Figure S1: Spearman correlation heatmap (color = ϱ; full matrix shown) with numeric Spearman ϱ values in cells (n = 73). Correlations were computed on creatinine-normalized urinary concentrations (µmol/g creatinine; NAD in nmol/g creatinine) and derived indices (IDO index = KYN/TRP × 1000; QUIN/KYNA); Table S3: De-identified individual urinary marker values with key covariates (age at sampling, sex, spot creatinine); sequential participant IDs; n = 73; Table S4: Sex-stratified comparisons (Mann–Whitney U; false discovery rate (FDR)). q denotes BH-FDR-adjusted p-values across the variables listed in this table; Table S5: Neopterin–KP associations in creatinine-normalized ratios vs reconstructed absolute concentrations, including partial correlations adjusting for creatinine; Table S6: Neopterin status subgroup comparisons in reconstructed absolute units and spot creatinine; Table S7: Exploratory age–marker associations (Spearman; partial Spearman controlling age); Table S8: Age- and sex-adjusted rank-based linear models in reconstructed absolute units (OLS on z-scored ranks; bootstrap 95% CI, 1,000 resamples); Figure S2: Age associations for urinary neopterin and QUIN (n = 73); Note S1: Sensitivity analyses excluding potentially dilute creatinine samples.

Table S1. LC–MS/MS MRM transitions and parameters provided by the service laboratory (SCIEX TripleQuad 5500+, scheduled MRM).

Analyte	Polarity	Q1 (m/z)	Q3 (m/z)	DP	CE	RT (min)	Internal standard
Tryptophan	ESI+	205.2	188.1	72	31	3.12	Tryptophan-d5
Kynurenine	ESI+	209.1	192.1	80	13	2.85	Kynurenine-d3
3-Hydroxykynurenine	ESI+	225.1	110.0	87	27	1.45	3-Hydroxykynurenine-d4
Kynurenic acid	ESI+	190.2	144.1	101	50	3.38	Kynurenic acid-d5
NAD+	ESI+	664.3	136.1	145	50	1.52	(NAD+)-13C5
Quinolinic acid	ESI−	166.1	78.1	−55	−20	1.03	Quinolinic acid-d3
Neopterin	ESI+	254.1	206.1	78	26	0.89	2-picolinic acid-d4

Table S2. Analytical performance summary provided by the service laboratory (LOD/LOQ, linear range, intra-assay precision).

Analyte	Unit	LOD (3× S/N)	LOQ (6× S/N)	Linear range	Intra-assay CV (%)
Tryptophan	µmol/L	0.155	0.310	0–122	3.79
L-Kynurenine	µmol/L	0.007	0.013	0–12	3.65
Quinolinic acid	µmol/L	0.069	0.137	0–120	3.36

Analyte	Unit	LOD (3× S/N)	LOQ (6× S/N)	Linear range	Intra-assay CV (%)
Kynurenic acid	µmol/L	0.015	0.030	0–52.8	5.12
3-Hydroxykynurenine	µg/L	6.250	12.500	0–5000	8.00
Neopterin	µg/L	0.325	0.650	0.65–2500	8.36

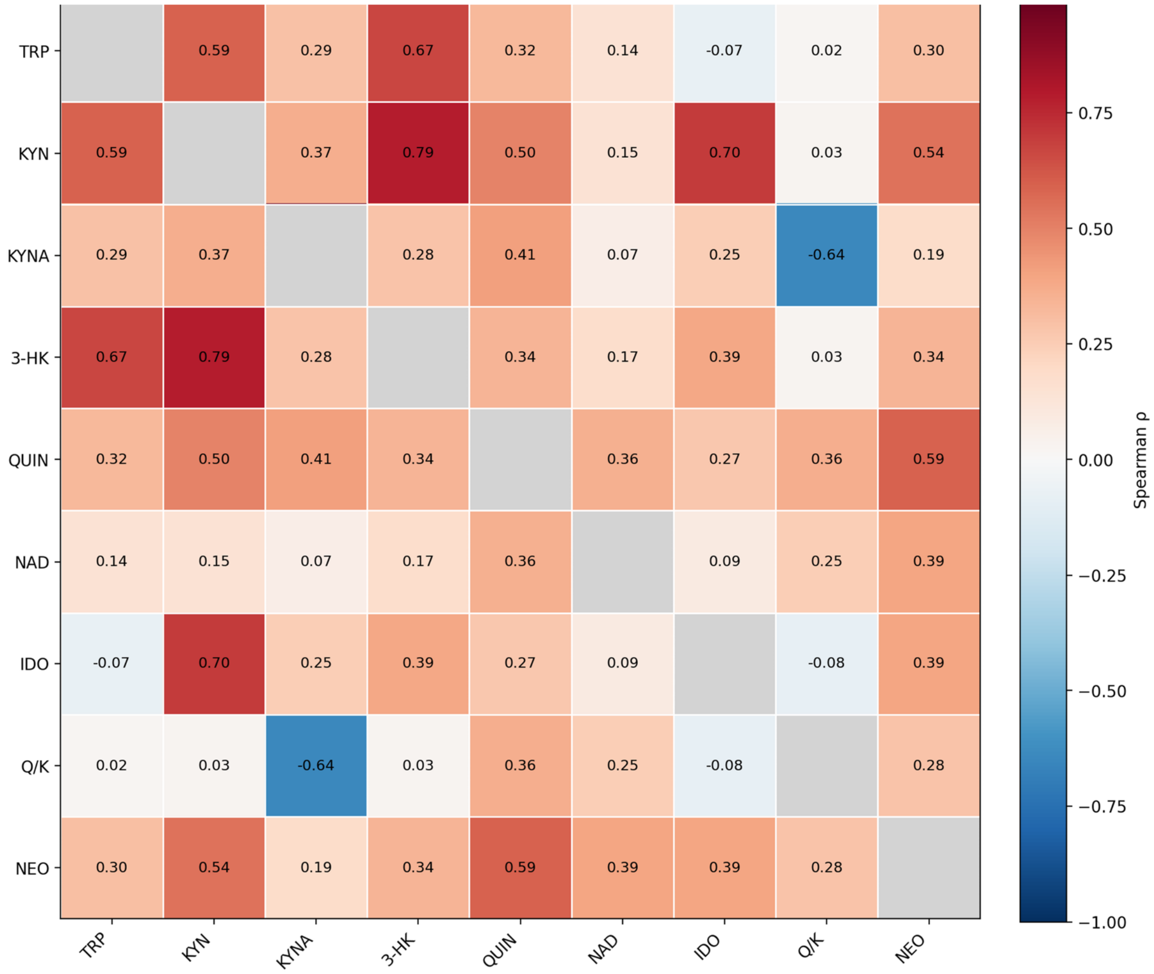

Figure S1. Spearman correlation heatmap (color = ϱ; full matrix shown) with numeric Spearman ϱ values in cells (n = 73). Correlations were computed on creatinine-normalized urinary concentrations (µmol/g creatinine; NAD in nmol/g creatinine) and derived indices (IDO index = KYN/TRP × 1000; QUIN/KYNA).

Table S3. De-identified individual urinary marker values with key covariates (age at sampling, sex, spot creatinine); sequential participant IDs; n = 73.

Participant_ID	Tryptophan (µmol/g creatinine)	Kynurenine (µmol/g creatinine)	Kynurenic acid (µmol/g creatinine)	3-OH-Kynurenine (µmol/g creatinine)	Quinolinic acid (µmol/g creatinine)	NAD (nmol/g creatinine)	IDO index (KYN/TRP×1000) (−)	QUIN/KYNA ratio (−)	Neopterin (µmol/g creatinine)	Creatinine (mg/L)	Sex (M-male/F-female)	Age (years)
P01	108.65	5.77	11.83	4.17	63.7	107.8	53.1	5.39	2.3	1141.0	M	6
P02	83.45	2.88	7.59	1.23	36.21	74.5	34.5	4.77	1.67	1450.0	M	12
P03	62.26	3.34	9.82	1.74	96.16	133.8	53.6	9.8	2.74	398.0	M	4
P04	66.02	4.34	8.46	1.75	50.05	79.0	65.7	5.92	2.68	645.0	M	7
P05	115.2	4.75	7.86	2.18	50.02	111.6	41.2	6.37	3.38	666.0	M	8
P06	132.54	17.01	19.35	5.53	72.97	159.6	128.3	3.77	3.09	418.0	M	3
P07	89.25	3.96	20.17	2.53	50.31	43.4	44.4	2.49	1.14	932.0	M	7
P08	62.28	3.59	17.52	1.23	77.72	53.5	57.7	4.44	3.53	1010.0	F	6
P09	117.63	11.96	13.69	3.14	60.06	47.4	101.6	4.39	4.82	363.0	M	5
P10	87.19	7.48	19.09	2.67	100.83	105.0	85.8	5.28	5.45	242.0	F	5
P11	61.89	3.9	14.92	0.61	66.8	155.3	63.0	4.48	5.66	244.0	M	3
P12	94.2	5.44	13.7	1.9	50.58	93.5	57.8	3.69	2.24	1380.0	F	10
P13	63.49	5.71	12.86	1.69	52.49	60.8	90.0	4.08	1.93	1890.0	M	10
P14	79.43	4.12	12.23	1.19	56.43	60.4	51.8	4.61	1.81	891.0	F	3
P15	153.38	14.06	22.42	3.99	84.7	124.2	91.6	3.78	3.13	281.0	M	5
P16	42.86	2.66	10.81	1.59	48.28	135.7	62.1	4.46	1.92	406.0	M	5
P17	34.29	3.64	21.19	1.05	48.24	91.7	106.2	2.28	2.99	939.0	M	9
P18	42.77	2.91	16.06	1.29	48.03	105.6	68.0	2.99	1.08	723.0	M	4
P19	28.28	3.42	11.41	0.93	23.79	54.3	121.0	2.09	1.86	824.0	M	5
P20	58.05	2.65	10.26	1.3	53.86	82.7	45.7	5.25	2.31	739.0	M	4
P21	116.89	13.26	15.53	3.48	46.69	111.7	113.5	3.01	3.34	578.0	M	3
P22	45.95	3.14	5.24	1.35	43.08	79.9	68.3	8.22	1.28	1428.0	M	4
P23	73.23	5.98	20.52	1.78	78.06	86.1	81.7	3.8	3.67	373.0	F	3
P24	61.68	5.98	8.04	3.27	39.38	79.7	96.9	4.9	2.59	843.0	M	5
P25	38.72	4.02	9.23	1.18	22.56	25.0	103.8	2.44	0.42	1170.0	M	13
P26	94.04	2.99	13.22	1.79	52.19	67.5	31.8	3.95	1.22	542.0	M	7
P27	71.21	1.51	12.38	0.84	67.89	171.4	21.1	5.48	1.73	601.0	M	3
P28	71.82	3.3	13.47	1.24	49.57	29.6	45.9	3.68	0.97	652.0	M	7
P29	59.39	2.04	9.39	0.77	41.23	94.4	34.3	4.39	1.77	958.0	M	10
P30	54.65	3.53	25.21	1.37	67.62	82.2	64.5	2.68	4.2	686.0	F	4
P31	48.79	3.24	13.66	1.38	70.78	281.7	66.4	5.18	2.59	1164.0	M	3
P32	134.92	9.52	18.74	3.04	53.36	59.7	70.6	2.85	1.66	461.0	M	8
P33	112.38	4.97	11.69	2.2	61.81	97.6	44.2	5.29	3.16	864.0	F	5
P34	68.17	6.65	29.19	2.47	57.25	116.8	97.5	1.96	2.9	531.0	M	7
P35	149.08	15.42	14.09	3.84	71.15	121.3	103.4	5.05	3.39	973.0	M	5

P36	56.74	5.73	9.61	2.2	66.85	93.0	101.0	6.96	1.64	356.0	M	3
P37	66.81	2.63	11.14	0.99	55.31	94.3	39.4	4.97	1.6	687.0	F	7
P38	41.8	2.37	8.57	1.85	53.92	117.5	56.7	6.29	1.6	701.0	M	6
P39	57.58	2.65	10.0	1.2	42.95	68.0	46.1	4.3	1.3	1320.0	M	10
P40	51.65	4.68	6.16	1.77	65.02	106.0	90.6	10.55	2.36	972.0	M	5
P41	88.34	5.54	14.27	3.12	61.34	93.8	62.7	4.3	1.95	626.0	M	5
P42	70.27	9.58	10.9	5.35	96.4	90.1	136.3	8.84	7.57	333.0	M	5
P43	73.41	2.7	10.37	0.34	49.66	81.8	36.8	4.79	2.37	737.0	M	4
P44	118.71	4.75	15.59	2.64	61.49	50.2	40.0	3.94	2.29	795.0	M	4
P45	54.27	1.85	11.11	1.09	40.05	31.5	34.1	3.61	0.76	1990.0	M	13
P46	30.84	4.37	16.6	1.04	53.94	75.9	141.7	3.25	1.25	723.0	F	5
P47	82.78	4.77	12.41	2.43	71.87	72.4	57.6	5.79	1.06	935.0	M	6
P48	76.89	1.89	18.02	1.85	44.91	100.9	24.6	2.49	1.48	688.0	F	4
P49	58.47	0.96	6.73	0.65	41.75	74.5	16.4	6.2	1.04	1370.0	M	4
P50	69.3	4.63	10.37	1.96	54.7	147.1	66.8	5.27	2.87	918.0	M	3
P51	68.26	3.19	8.72	1.67	47.78	12.5	46.8	5.48	0.92	586.0	M	4
P52	67.72	4.9	11.16	1.28	66.87	68.6	72.3	5.99	4.73	235.0	F	4
P53	70.35	3.16	13.19	1.38	40.88	81.2	44.9	3.1	1.5	1130.0	M	10
P54	92.98	3.01	8.27	1.62	30.38	50.8	32.4	3.67	1.41	1366.0	M	12
P55	60.22	3.22	10.48	1.55	50.65	58.1	53.4	4.83	1.75	1860.0	M	5
P56	91.83	5.41	20.01	2.57	47.7	101.6	58.9	2.38	0.89	925.0	F	7
P57	83.4	4.91	16.19	2.01	61.37	88.7	58.8	3.79	3.14	976.0	M	4
P58	110.4	3.43	5.85	2.37	44.21	106.7	31.1	7.56	2.78	891.0	M	6
P59	43.48	2.43	9.11	2.02	34.96	91.9	55.9	3.84	2.71	135.0	F	4
P60	80.28	5.5	15.09	2.32	51.38	208.7	68.6	3.4	1.56	218.0	M	3
P61	77.67	5.59	10.15	2.03	70.36	78.6	72.0	6.93	2.58	671.0	M	3
P62	124.91	7.39	11.31	2.82	83.04	123.8	59.2	7.34	2.79	865.0	F	3
P63	63.75	3.61	10.2	1.07	96.71	123.7	56.6	9.48	3.68	1520.0	M	3
P64	104.35	8.02	3.63	2.21	50.38	120.9	76.9	13.87	5.28	697.0	M	3
P65	60.87	2.73	8.11	0.83	41.42	146.5	44.9	5.11	1.63	1270.0	M	9
P66	69.67	3.16	6.86	1.21	57.97	110.9	45.4	8.46	1.99	1794.0	M	4
P67	112.92	13.11	14.54	2.92	71.54	119.4	116.1	4.92	5.06	650.0	M	4
P68	94.69	6.1	16.26	2.39	160.47	92.3	64.4	9.87	3.39	941.0	M	6
P69	74.16	1.91	16.58	0.89	61.15	108.0	25.8	3.69	2.03	538.0	F	4
P70	77.59	4.56	18.24	2.22	59.83	88.0	58.8	3.28	2.31	839.0	M	5
P71	99.5	8.7	10.29	2.97	49.69	78.4	87.5	4.83	2.21	795.0	F	7
P72	34.61	2.32	7.56	0.87	43.48	72.5	67.1	5.75	1.77	1150.0	M	7
P73	52.04	3.71	10.75	1.44	54.39	56.0	71.4	5.06	2.07	638.0	M	6

Table S4. Sex-stratified comparisons (Mann–Whitney U; false discovery rate (FDR)). q denotes BH-FDR-adjusted p-values across the variables listed in this table.

Variable	Median (M)	Median (F)	p	q (FDR)	Cliff's δ (M vs F)
Tryptophan (μmol/g creatinine)	69.67	75.525	0.553	0.836	-0.099
Kynurenine (μmol/g creatinine)	3.9	4.635	0.79	0.836	-0.045
Kynurenic acid (μmol/g creatinine)	10.9	15.14	0.012	0.115	-0.417
3-OH-Kynurenine (μmol/g creatinine)	1.75	1.815	0.759	0.836	0.052

Quinolinic acid (µmol/g creatinine)	52.49	58.79	0.172	0.484	-0.226
NAD (nmol/g creatinine)	90.1	92.7	0.836	0.836	0.035
IDO index (—)	62.7	58.35	0.674	0.836	0.07
QUIN/KYNA ratio (—)	4.83	4.14	0.194	0.484	0.215
Neopterin (µmol/g creatinine)	2.07	2.475	0.298	0.597	-0.172
Creatinine enzymatic (mg/L)	824	705.5	0.178	0.484	0.223

Table S5: Neopterin–KP associations in creatinine-normalized ratios vs reconstructed absolute concentrations, including partial correlations adjusting for creatinine. Spearman ϱ values are shown; q36 values are Benjamini–Hochberg false discovery rate (FDR)-adjusted across the 36 unique pairwise tests of the full 9-variable correlation matrix for each representation (creatinine-normalized, reconstructed absolute, and partial). Dashes indicate derived indices not evaluated in the reconstructed-absolute and partial columns.

Marker	Creatinine-normalized (µmol/g or nmol/g) (Spearman ϱ, q36)	Reconstructed absolute (µmol/L or nmol/L) (Spearman ϱ, q36)	Partial (abs ∣ creatinine) (Spearman ϱ, q36)
TRP	ϱ = 0.30, q36 = 0.018	ϱ = 0.58, q36 = 2.49 × 10^{-7}	ϱ = 0.29, q36 = 0.026
KYN	ϱ = 0.54, q36 = 3.69 × 10^{-6}	ϱ = 0.61, q36 = 3.72 × 10^{-8}	ϱ = 0.46, q36 = 2.87 × 10^{-4}
3-HK	ϱ = 0.34, q36 = 0.007	ϱ = 0.54, q36 = 1.84 × 10^{-6}	ϱ = 0.33, q36 = 0.013
KYNA	ϱ = 0.19, q36 = 0.156	ϱ = 0.40, q36 = 6.61 × 10^{-4}	ϱ = 0.01, q36 = 0.930
QUIN	ϱ = 0.59, q36 = 2.64 × 10^{-7}	ϱ = 0.68, q36 = 2.69 × 10^{-10}	ϱ = 0.46, q36 = 2.52 × 10^{-4}
NAD	ϱ = 0.39, q36 = 0.002	ϱ = 0.64, q36 = 5.87 × 10^{-9}	ϱ = 0.40, q36 = 0.002
IDO index	ϱ = 0.39, q36 = 0.002	—	—
QUIN/KYNA ratio	ϱ = 0.28, q36 = 0.026	—	—

Table S6. Neopterin status subgroup comparisons in reconstructed absolute units and spot creatinine.

Analyte	Unit	Neopterin >2 (median (IQR))	Neopterin ≤2 (median (IQR))	p	q	Cliff's δ
Creatinine	mg/L	717.00 (502.75–923.25)	925.00 (652.00–1320.00)	0.022	0.154	-0.31
TRP	µmol/L	54.75 (37.17–79.68)	56.90 (40.00–80.10)	0.678	0.791	-0.06
KYN	µmol/L	3.77 (2.41–5.51)	3.47 (1.87–4.18)	0.083	0.291	0.24
3-HK	µmol/L	1.57 (0.96–2.03)	1.27 (0.81–1.95)	0.502	0.791	0.09
KYNA	µmol/L	8.13 (5.40–14.11)	10.30 (7.65–12.31)	0.2	0.467	-0.18
QUIN	µmol/L	39.45 (31.70–55.03)	40.80 (30.90–52.60)	0.907	0.907	-0.02
NAD	nmol/L	63.45 (48.19–86.64)	69.39 (45.50–93.98)	0.662	0.791	-0.06

Table S7. Exploratory age–marker associations (Spearman; partial Spearman controlling age)

Association (*n* = 73)	ϱ	p
Spearman ϱ (Neopterin (µmol/g creatinine), age)	-0.37	0.001
Spearman ϱ (QUIN (µmol/g creatinine), age)	-0.47	3.26 × 10^{-5}
Partial ϱ (Neopterin (µmol/g creatinine), QUIN (µmol/g creatinine) ∣ age)	0.50	5.24 × 10^{-6}

Partial ϱ (Neopterin (μmol/g creatinine), KYN (μmol/g creatinine) ∣ age)	0.53	1.69×10^{-6}

Table S8. Age- and sex-adjusted rank-based linear models in reconstructed absolute units (OLS on z-scored ranks; bootstrap 95% CI, 1000 resamples).

Outcome (absolute units)	Predictor	Adjustments	Standardized β	95% CI	p
QUIN_abs (μmol/L)	Neopterin_abs	Age, sex	0.68	0.51–0.80	6.13×10^{-11}
KYN_abs (μmol/L)	Neopterin_abs	Age, sex	0.60	0.41–0.74	1.69×10^{-8}

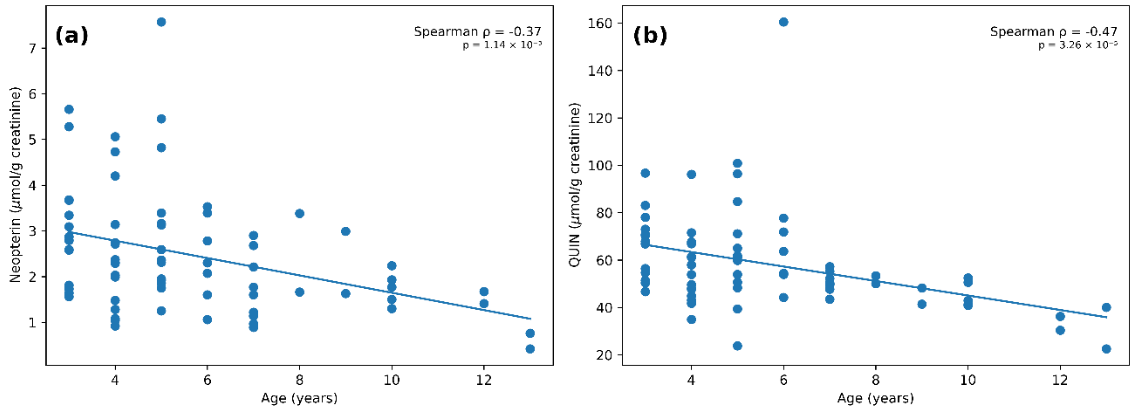

Figure S2. Age associations for urinary neopterin and QUIN (*n* = 73): (**a**) neopterin vs age; (**b**) QUIN vs age. Scatter plots shown with least-squares fit; Spearman ϱ and p are reported in Table S7.

Note S1. Sensitivity analyses excluding potentially dilute creatinine samples.

Sensitivity analyses were performed by excluding samples with creatinine below the laboratory-provided lower reference limit (400 mg/L; *n* = 11). Key correlation results were unchanged in direction and remained statistically significant; for example, the neopterin–QUIN association remained strong (Spearman ϱ = 0.55, p = 4.01×10^{-6}). Group comparisons by neopterin status in creatinine-normalized units also remained consistent for KYN, 3-HK, QUIN, TRP, IDO index (KYN/TRP × 1000), and NAD (all q<0.05 after FDR correction), whereas QUIN/KYNA ratio and KYNA did not reach significance after FDR correction (Table 3). Because spot creatinine differed between neopterin subgroups, we additionally reconstructed absolute concentrations and repeated subgroup comparisons (Table S6). Results were also robust to alternative handling of the single NAD value reported as <LOQ. The neopterin–NAD correlation was similar when using ½LOQ imputation (12.5 nmol/g creatinine; Spearman ϱ = 0.39, p = 5.85×10^{-4}), substituting the LOQ value (25.0 nmol/g creatinine; ϱ = 0.39, p = 5.84×10^{-4}), or excluding the left-censored observation (*n* = 72; ϱ = 0.37, p = 1.43×10^{-3}).
